# Transcriptomics in human blood incubation reveals the importance of oxidative stress response in *Saccharomyces cerevisiae* clinical strains

**DOI:** 10.1186/1471-2164-13-419

**Published:** 2012-08-23

**Authors:** Silvia Llopis, Amparo Querol, Antje Heyken, Bernhard Hube, Lene Jespersen, M Teresa Fernández-Espinar, Roberto Pérez-Torrado

**Affiliations:** 1 Instituto de Agroquímica y Tecnología de los Alimentos, IATA-CSIC, P.O. Box, 73. E-46100, Burjassot, Spain; 2 Department of Microbial Pathogenicity Mechanisms, Leibniz Institute for Natural Product Research and Infection Biology, Hans-Knoell-Institute (HKI), Jena, Germany; 3 Friedrich Schiller University, Jena, Germany; 4 Department of Food Science, Food Microbiology, Faculty of Life Sciences, University of Copenhagen, Copenhagen, Denmark

**Keywords:** *Saccharomyces cerevisiae*, Clinical strains, Transcriptomics, Blood, Oxidative stress

## Abstract

**Background:**

In recent years an increasing number of yeast infections in humans have been related to certain clinical isolates of *Saccharomyces cerevisiae*. Some clinical strains showed *in vivo* and *in vitro* virulence traits and were able to cause death in mice whereas other clinical strains were avirulent.

**Results:**

In this work, we studied the transcriptional profiles of two *S. cerevisiae* clinical strains showing virulent traits and two control non-virulent strains during a blood incubation model and detected a specific transcriptional response of clinical strains. This response involves an mRNA levels increase of amino acid biosynthesis genes and especially oxidative stress related genes. We observed that the clinical strains were more resistant to reactive oxygen species *in vitro*. In addition, blood survival of clinical isolates was high, reaching similar levels to pathogenic *Candida albicans* strain. Furthermore, a virulent strain mutant in the transcription factor Yap1p, unable to grow in oxidative stress conditions, presented decreased survival levels in human blood compared with the wild type or *YAP1* reconstituted strain.

**Conclusions:**

Our data suggest that this enhanced oxidative stress response in virulent clinical isolates, presumably induced in response to oxidative burst from host defense cells, is important to increase survival in human blood and can help to infect and even produce death in mice models.

## Background

*S. cerevisiae* is a ubiquitous organism that can be found in nature on plants, fruits and in soil. This species is involved in fermentative processes in beer, bread and wine, and is even consumed as a nutritional supplement, always being traditionally regarded as absolutely safe. *S. cerevisiae* and its commercially available preparations known as *Saccharomyces boulardii*, that are used to treat antibiotic-related diarrhea, have recently been shown to have the potential to cause a wide variety of infections, ranging from cutaneous infections and vaginitis in healthy patients to systemic infections of the bloodstream and vital organs in immunocompromised and critically ill patients [1-3]. The patients infected are mainly premature children, elderly people or patients suffering from immunosuppression due to AIDS, treatment with immunosuppressive agents, and other conditions associated with an insufficient immune response. Moreover, severe infections by *S. cerevisiae* have been occasionally reported in patients with no obvious predisposing factors [4,5].

Some *S. cerevisiae* clinical strains have been isolated from blood [4,6-8]. The main routes for bloodstream infections are probably translocation of ingested yeast from the gut or direct contamination of the central venous catheter insertion site [9-11]. Similarly to other opportunistic fungal pathogens such as *C. glabrata*, the ability to colonize and cause disease in the host depends on the immune status of the host and the expression of certain virulence factors by the pathogen [12,13]. The majority of *S. cerevisiae* clinical isolates secrete high levels of proteases and phospholipases, can grow at 42°C, exhibit multiple colony phenotypes, have ability to adhere to epithelial tissue and show invasive pseudohyphal growth on solid agar [9,14-16]. These studies showed that clinical isolates differed phenotypically from laboratory and wine strains *in vitro*, but it remains unclear whether these traits also play a role during infection. Comparison of *S. cerevisiae* clinical and non-clinical strains by molecular typing and determination of putative virulence traits has not revealed a specific virulence factor that clearly separates the strains of clinical origin from the strains of non-clinical origin [17,18]. In addition, little is known about the interactions between *S. cerevisiae* and host defence cells or non-cellular components [18].

mRNA level profiles of clinical strains of *S. cerevisiae* following exposure to whole blood rather than serum may closely reflect the *in vivo* response of these yeasts during bloodstream infections, since *C. albicans* expression profiles following intravenous infection of mice closely resembled profiles following incubation in whole blood [19], which in turn strongly correlated with the expression profile of *C. albicans* exposed to neutrophils [20]. In these studies, the authors identified differentially expressed *C. albicans* genes involved in general stress response, antioxidative response, the glyoxylate cycle as well as putative virulence genes expressed in response to human blood cells [19].

To better understand the physiology of virulent *S. cerevisiae* cells during infection, we performed a transcriptional analysis to investigate which genes and pathways are required for survival in blood as a model of human bloodstream infection. We compare two control non-virulent strains with two virulent isolates (60 and D14). These strains showed the highest levels in virulence factors, were able to colonize immunocompetent mice brain after blood infections and cause mice death, showing increased adherence ability whereas other clinical isolates showed low virulence levels [3,18]. Comparison of control and virulent strains revealed that amino acid biosynthesis and elevated oxidative stress response can be of special relevance for survival of *S. cerevisiae* virulent strains to host defences. Phenotypic analysis of virulent strains and a *YAP1* mutant, unable to respond to oxidative stress, confirmed the importance of the increased oxidative stress response for yeast survival in human blood incubations.

## Results

### Transcription profile of yeasts in human blood

To obtain a global view of the *S. cerevisiae* response to blood environment and to investigate which set of genes are expressed in the different yeast, we analyzed the transcription profile of avirulent (W303 and CECT 10431) and virulent (D14 and 60) strains exposed to blood. We incubated yeast cells in human blood using the model previously established by Fradin et al. [19] and searched for significantly over-represented functional groups in up-regulated (Table 1) or in the down-regulated (Table 2) set of genes during the time course of the experiment (Genes and significances are presented in Additional file 1: Table S1 and Table S2). A complete microarray dataset is presented in Aditional file 2: Table S3.

**Table 1:** **Significant functional groups observed in up-regulated genes after 15, 30 and 60 min of incubation in blood for virulent (60, D14) and non-virulent (CECT 10431, W303) strains**^**a**^

**Functional groups**	**Time (min)**											
	**15**				**30**				**60**			
	**60**	**CECT 10431**	**W303**	**D14**	**60**	**CECT 10431**	**W303**	**D14**	**60**	**CECT 10431**	**W303**	**D14**
Translation	+	+	-	+	+	+	-	+	+	+	-	+
Ribosome biogenesis	+	+	-	+	+	+	-	+	+	+	+	+
Gene expression	+	+	-	+	+	-	-	+	+	+	-	+
Amino acid biosynthetic process	+	-	-	-	+	+	-	+	+	+	-	+
Aspartate family amino acid biosynthetic process	-	-	-	-	+	+	-	+	+	-	-	-
Arginine biosynthetic process	-	-	-	-	+	+	-	+	+	-	-	-

^a^Functional groups were (+) or were not (−) significantly overrepresented (*p*-values < 0.05).

**Table 2:** **Functional groups observed in down-regulated genes after 15, 30 and 60 min of incubation in blood for virulent (60, D14) and non-virulent (CECT 10.431, W303) strains**^**a**^

**Functional groups**	**Time**											
	**15**				**30**				**60**			
	**60**	**CECT 10431**	**W303**	**D14**	**60**	**CECT 10431**	**W303**	**D14**	**60**	**CECT 10431**	**W303**	**D14**
Protein folding	-	+	+	-	-	-	+	-	-	+	-	-
Oxidative phosphorylation	-	-	-	-	-	+	-	-	-	+	-	-
Glutamate biosynthetic process	-	-	-	-	-	-	-	-	-	-	-	+

^a^Functional groups were (+) or were not (−) significantly overrepresented (*p*-values < 0.05).

YPD precultured cells were preadapted in PBS buffer before inoculation in human blood [19]. Preadapted cell suspension was inoculated into fresh whole human blood and incubated for 0, 15, 30 and 60 min at 37°C. mRNA levels was determined by microarray hybridization. The arrays were hybridized with each time point and the time point 0 min for each strain, thus, mRNA level data for 15, 30 and 60 min reflects the induction respect time 0. Data were ratio-based normalized and a FDR of 5% was used to identify statistically significant data. The microarray transcriptomic data presented corresponds to the average of three biological replicates including a dye swap. A complete set of data for all genes is included in Additional file 2: Table S3. Data was validated measuring mRNA levels of 7 genes by qRT-PCR in different strains and conditions (Additional file 1: Table S4) showing that inductions or repressions differ less than 20% between the two methods.

First, we searched in the data for significantly represented functional groups in up-regulated (Table 1) or in the down-regulated (Table 2) set of genes (Bonferroni corrected *p*-values < 0.05). After 15 min of blood incubation, three functional groups (Translation, Biogenesis of ribosome and Gene expression) were found in the induced set of genes in all strains except for the laboratory strain W303. These significantly represented functional groups that comprise genes related with normal vegetative growth, appeared in the up-regulated genes during all the experiment, except time point 0 min, indicating a continuous adaptation of these strains to the blood environment. For strain W303, the functional group Ribosome biogenesis was significant only after 60 min of incubation, revealing a delayed adaptation to the new environment. Strains CECT 10431, 60 and D14 showed no differences in this set of functional groups. Genes belonging to these functional groups include ribosomal protein genes such as *RPL32* and *RPS10*, translational elongation factor genes such as *TEF2* and *EFB1*, and tRNA synthetase genes such as *THS1.*

The functional group Amino acid biosynthetic process was observed in strain 60 at time point 15 min and in strains 60, D14 and CECT 10431 after 30 and 60 min. Furthermore, the functional groups Aspartate family amino acid biosynthetic process and Arginine biosynthetic process were significant for strain 60, D14 and CECT 10431 at 30 min and for strain 60 at the last time point (60 min).

Some functional groups were found to be significantly repressed (Table 2). Protein folding was observed in CECT 10431 after 15 min of incubation and during the entire time course in W303, but not in the virulent strains 60 and 14. The main function of the genes included in this group, such as *SSA2*, is to encode molecular chaperones, which bind newly-translated proteins to assist proper folding and to prevent aggregation/misfolding [21]. The down regulation of genes of this functional group in W303 is consistent with the observed delay in the functional groups related to preparation for growth like Ribosome biogenesis. In the down-regulated set of genes, the functional group Oxidative phosphorylation was observed in strain CECT 10431 after 30 and 60 min and the functional group Biosynthesis of glutamate was detected at 60 min. No significant functional group was observed in the down-regulated genes in virulent strains 60 and D14.

On the other hand, we found significant changes in the mRNA levels of specific genes implicated in important biological process (see Additional file 2: Table S3). At the early stage (15 min), several genes encoding glycolytic enzymes were up-regulated. The genes encoding glucose-6-phosphate isomerase (*PGI1*), phosphofructokinase (*PFK2*) and aldolase (*FBA1*) were up-regulated at early stages of incubation, but the mRNA levels were even higher after 30 min and 60 min for wine strain CECT 10431 and D14 respectively. For strain 60, the maximal mRNA level was monitored after 60 min. For the laboratory strain W303, the mRNA level of all three genes was down-regulated at 15 min, *PGI1* was weakly induced at 30 min, and *PFK2* mRNA level was up-regulated at 60 min. In strain 60, other genes that encode glycolytic enzymes were mostly down-regulated in the early stages compared to 0 min time point, but transcripts were considerably elevated at the later stages of the incubation, such as, glyceraldehyde 3-phosphate dehydrogenase (*TDH3*), phosphoglycerate kinase (*PGK1*) and enolase (*ENO1*). Also, the mRNA level of gluconeogenic enzyme phosphoenolpyruvate carboxykinase (*PCK1*) was strongly down-regulated in this strain.

The genes encoding the key enzymes of the glyoxylate cycle, isocitrate lyase (*ICL1*) and malate synthase (*MLS1*) were down-regulated in all strains during the time course of the experiment, although this repression was less strong at the later stage in strains D14 and 60. These genes are subjected to catabolite repression which suggests that yeast cells have access to glucose during all the experiment.

### Comparison of mRNA levels between virulent and non-virulent strains in human blood

To identify genes specifically expressed in *S. cerevisiae* virulent isolates in blood, we analyzed the mRNA levels at different incubation times and we identified statistically significant data when comparing average of virulent strains group (60 and D14) data with average of non-virulent group (W303 and CECT 10431) data. Then we searched for functional groups of genes whose mRNA level was specifically associated with virulent strains of *S. cerevisiae* (Table 3).

**Table 3:** **Functional groups observed in up-regulated genes in virulent strains related to control strains after 15, 30 and 60 min of incubation in blood**^**a**^

**Functional groups**	**Time (min)**			**Genes**
	**15**	**30**	**60**	
Base-excision repair	+	-	-	*POL30, POL31, RAD27, APN1, OGG1*
Vacuole organization	+	+	-	*CMD1, VPS3, CUP5, VTC1, SFK1, VID24/ MD1/ VPS45/ TRX2/ TRX1/ VAC7/ TPM1/ VTC3/*
Xenobiotic transporter	+	-	-	*PDR5, SNQ2, PDR12*
Amino acid biosynthetic process/ Amine biosynthetic process/ Nitrogen compound biosynthetic process	-	+	+	*ARO4/ ARO3/ LYS4/ HIS1/ ARG5/ARG6/ SER3/ILV1/ SER33/ HIS5/ LYS1/ TRP3/ ARG1/ ARG8/ LEU9/ ORT1/ SAM4*
Amino acid metabolic process	-	+	+	*ADH5/ ARO4/ ARO3/ LYS4/ HIS1/ ARG5/ARG6/ SER3/ ILV1/ADH4/ SER33/ HIS5/ LYS1/ URA2/ TRP3/ MSE1/ ARG1/ ARG8/ LEU9/ ORT1/ SAM4/*
Phosphatase activity	-	+	+	*PTC3/ PHO3/ PHO5/ DPP1/ LPP1/ SDT1/ PHO12/ INP51/ SAP185/ SAC1/ PPZ1/ TSL1/ YMR087WTPS3/ FCP1/ YNL217W/ PHO12*
Transmembrane transporter activity	-	+	-	*CTP1/ GGC1/ ATP17/ GNP1/ PIC2/ HXT10/ VPS73/ ZRT1/ TPO2/ TNA1/ AVT1/ VMA5/ ZRT3/ YBT1/ AQY2/ SMF3/ ZRT2/ ATR1/ ITR2/ ORT1/ ODC2/ PDR12/ YMC1/ ANT1/ TPO3/*
Cell redox homeostasis	-	-	+	*TTR1/ TRX2/ TRX1/ TSA1/ GLR1/ YNL134C/ TPS1/ PGM2/ GRE2/ MXR1/ PDI1/ UBA1/ LYS20/ AHP1/ TKL1/TAL1/ ADH6/ CYS3*

^a^ Functional groups were (+) or were not (−) significantly overrepresented (*p*-values < 0.05).

After 15 min of blood incubation, three functional groups were significantly overrepresented in the up-regulated set of genes in virulent strains compared to non-virulent strains: Vacuole organization, Xenobiotic transporter and Base-excision repair. The functional group Vacuole organization, induced again after 30 min, includes genes related with biogenesis, protein-vacuolar targeting and vacuole fusion (*CUP5*, *VPS3* and *VTC1*). The Base-excision repair pathway removes lesions that result from exposure to endogenous or exogenous reactive oxygen species (ROS) [22] and includes genes such as *POL30* (DNA polymerase processivity factor), *RAD27* (5' flap endonuclease), *APN1* (DNA apurinic or apyrimidinic site lyase) and *OGG1* (purine-specific oxidized base lesion DNA N-glycosylase). The level of mRNA of these genes decreased along the experiment in virulent strains.

Seven functional groups more were enhanced at time points 30 or 60 min in virulent strains. Four closely related groups (Amino acid biosynthetic process, Amino acid metabolic process, Amine biosynthetic process and Nitrogen compound biosynthetic process) include genes that take part in the biosynthesis of some aminoacids, such as aromatic aminoacids (*ARO3*, *ARO4*), histidine (*HIS1*, *HIS5*), arginine (*ARG1*, *ARG5*, *ARG6, ARG8*), tryptophan (*TRP3*), lysine (*LYS1*, *LYS4*), leucine (*LEU9*), and serine (*SER3*, *SER33*). We wanted to know if the presence of several auxotrophies in the laboratory strain W303 could indirectly affect the appearance of functional groups related to aminoacid metabolism in this analysis. We repeated the data analysis comparing the two virulent strains (D14 and 60) against the wine strain CECT 10431, excluding W303 strain. The results showed once again the appearance of aminoacid metabolism functional groups as Cellular aminoacid metabolic process, containing similar genes as (*LEU9, SER3, ARG1, ARG8*). Then we concluded that W303 auxotrophies are not biasing the results, probably because the short time sampling, and that aminoacid metabolism related genes are highly activated in the clinical isolates. The fifth functional group, Phosphatase activity, includes genes such as *PHO3*, *PHO5* and *PHO12* that are expressed under low-phosphate conditions. It may indicate a response to phosphate starvation during blood incubation. Also, *PHO3* is able to hydrolyze thiamin phosphates in the periplasmic space, increasing cellular thiamine uptake. Another functional group is Transmembrane transporter activity which includes genes that take part in the mitochondrial transport (*CTP1*, *YHM1*), zinc transport (*ZRT1*) and amino acid transport (*GNP1*).

**Figure 1:**
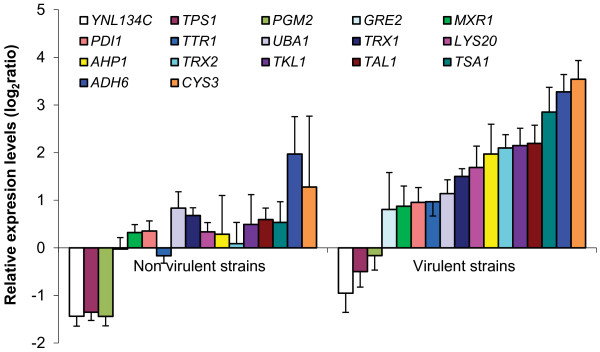
**Expression levels of oxidative stress related genes comparing virulent and non-virulent strains after 60 min of incubation in human blood.** Induction of oxidative stress related genes in non-virulent (CECT 10431 and W303) or virulent strains (D14 and 60) was averaged. All genes showed significantly different (*p*<0.05) values between the two groups using Student’s t-test statistical analysis. Average and standard errors 6 are shown.

A key functional group, Cell redox homeostasis, was found to be enhanced during the incubation in blood in virulent strains. The genes belonging to this group help to maintain a reduced environment in the cell and are associated with virulence in bacteria [23-25]. These genes play a significant role in defending the cell against oxidative stress exerted by reactive oxygen species (ROS), integral effectors of mammalian host defenses. Stimulated leucocytes can be a source of ROS, which may cause oxidative stress. Defense against this stress seems to be enhanced at 60 min of incubation of blood. Indeed, when we observed oxidative stress related genes, most of them (17) show statistically significant higher levels in virulent strains compared to non-virulent strains (Figure 1). On average, a very significant (p < 0.005) increase (0.20 to 1.43) is observed in the virulent strains respect to non-virulent strains. Interestingly, all the 17 genes are regulated by Yap1p, the main regulator of oxidative stress in yeasts (http://www.yeastract.com). Several components of the thioredoxin system are included in this set of genes. A thioredoxin peroxidase (*TSA1)* and two thioredoxin genes (*TRX1* and *TRX2*) were strongly upregulated in virulent strains at time 60 min. Also mRNA levels of *TRR1*, a thioredoxin-disulfide reductase gene, increase during the incubation in blood in strains 60 and D14.

Only the Pyridoxine metabolic process functional group was downregulated with regards to non-virulent strains at time point 30 min. This group includes genes (*SNZ*, *SNO2*) involved in pyridoxine metabolism and thiamine biosynthesis.

### Resistance to oxidative stress of virulent and non-virulent strains

**Figure 2:**
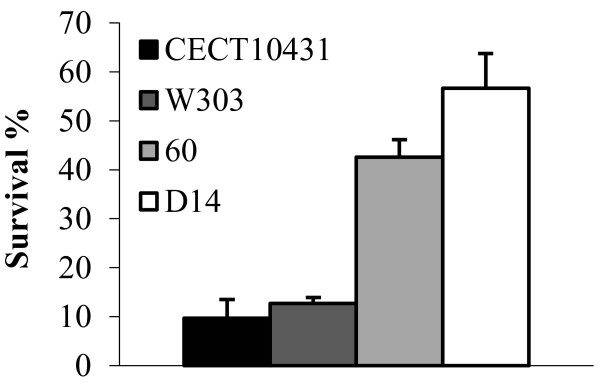
**Survival percentages comparison of virulent and non-virulent strains after oxidative stress.** The different strains were exposed to 6 mM HO for 1 hour and then plated in YPD media for colony counting. Percentages were calculated comparing with untreated cells. Averages and standard errors of independent triplicates are shown.

To confirm the relevance of the oxidative stress response on the virulent nature of yeast strains we measured the percentage of survival of these four strains after one hour in the presence of elevated levels (6 mM) of H_2_O_2_ (Figure 2). Virulent isolates exhibited higher survival rates whereas W303 and CECT 10431 showed very low survival. Statistical analysis confirmed significant differences in oxidative stress between virulent and non-virulent strains (p < 0.0005) whereas no significant differences between CECT 10431 and W303 or 60 and D14 were found (Additional file 1: Table S5).

### Survival of yeast strains in human blood

The aim of this experiment was to study the survival of control and pathogenic *S. cerevisiae* strains comparing them with *C. albicans* and also to know which blood cell types are affecting more yeast self-preservation. In this experiment *S. boulardii* strain Ultralevure, a bio-therapeutic agent which has been described as a potential pathogenic [6,9,10], was included. To investigate the survival of the different strains, we incubated the cells 10 min and 1 hour at 37°C in human blood (Table 4).

**Table 4:** **Yeasts survival in human blood incubations**^**a**^

**Species**	***C. albicans***	***S. cerevisiae***	***S. cerevisiae***	***S. boulardii***	***S. cerevisiae***	***S. cerevisiae***
**Strain**	**SC5314**	**60**	**D14**	**Ultralevure**	**CECT 10431**	**W303**
Time (min)						
10	87.4 ± 8.7	98.8 ± 8.7	76.5 ± 13.8	76.0 ± 13.8	-	66.0 ± 11.2^*^
60	56.4 ± 3.7	56.4 ± 3.7	60.5 ± 5.8	60.0 ± 5.8	50.8 ± 9.8	35.7 ± 4.8^*^

^a^ Survival is expressed as% of cfu respect time 0. *Strains individually compared to *C. albicans* SC5314 strain presented *p* < 0.05.

We observed that after 10 min the virulent strains D14 and 60 showed survival levels similar to the pathogenic *C. albicans* strain SC5314. In contrast, control strain W303 presented significantly lower survival. After 60 min of incubation the survival of all strains decreased. However we observed again a similar pattern since strains 60 and D14 showed comparable values to virulent SC5314 whereas W303 presented significantly lower survival. *S. boulardii* strain showed similar values to the *C. albicans* strain in this assay. The wine strain CECT10431 also presented survival levels lower than *C. albicans* and virulent *S. cerevisiae* strains but data was not significant.

### A virulent strain mutant in Yap1p showed decreased survival in human blood

**Figure 3:**
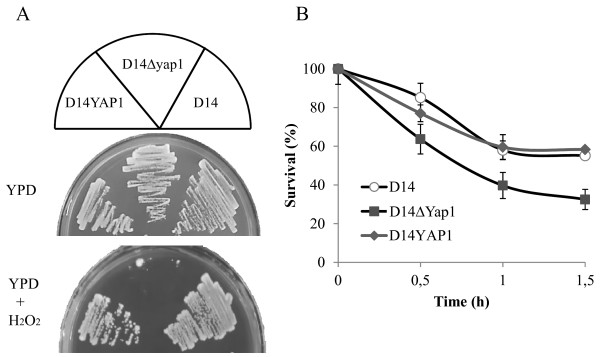
**Implication of Yap1p transcription factor in human blood yeast survival.** In panel **A**, the strain D14 and a *YAP1* mutant were plated in YPD or YPD with 2.5 mM HO. In panel **B**, blood survival assay is shown for wild type D14 and *YAP1* mutant strain. Yeasts were inoculated 1:1 ratio respect leucocytes and yeast survival was followed for 1.5 hours by plating in YPD agar plates. Data corresponds to average and standard errors of three independent biological replicates.

To confirm that oxidative stress response of virulent strains is important to survive in human blood we decided to study performance of a strain unable to grow in oxidative stress conditions. Since all genes with increased mRNA levels in virulent strains observed in Figure 1 are regulated by the transcription factor Yap1p, we decided to study survival in human blood comparing the wild type strain D14 with a homozygous for a Δ yap1 mutation. First we confirmed that D14Δyap1 was unable to survive in the presence of oxidative stress conditions (2.5 mM H_2_O_2_) whereas the wild type strain presented no growth defects (Figure 3A). Reincorporation of *YAP1* in D14YAP1 strain reverted this phenotype. This experiment confirmed that Yap1p is essential to perform a proper oxidative stress response in virulent strains. Then we evaluated the behavior of the D14Δyap1 strain in human blood incubation assay. Using the same conditions described above, yeast cells were added to blood and survival was determined after 0, 30, 60 and 90 min (Figure 3B). The results showed that D14Δyap1 survival decreased significantly (*p*-value < 0.05 at 30 or 60 and p-value < 0.01 at 90 min time points) reaching a level comparable to that of the laboratory strain W303 at 60 min (Table 4), confirming the relevance of a proper oxidative stress response to survive in human blood. Again, D14YAP1 showed similar phenotype than the wild type, confirming the involvement of Yap1p in blood survival.

## Discussion

Blood is a complex milieu composed of several types of immunoactive cells and molecules. As we have previously observed, several clinical strains of *S. cerevisiae* adapt to blood environment and are able to survive and spread to other tissues in murine models. To better understand the response of *S. cerevisiae* to this complex environment and to search for special features of virulent strains we have analysed the transcriptome of *S. cerevisiae* cells after contact with human blood. The results showed that they express several groups of genes in order to rapidly accommodate to this special environment. As expected, a high mRNA level of genes encoding factors involved in protein synthesis were detected in every strain after 15 min inoculation in blood, except for the laboratory strain W303. The strong induction of these set of genes is probably a consequence of the yeast cells transfer from nutrient poor medium (PBS) to a relatively nutrient-rich medium (blood). The downregulation of *PCK1*, encoding the gluconeogenic enzyme phosphoenolpyruvate carboxykinase, and other genes supports that yeast cells are exposed to a carbohydrate-rich environment, even at the later stages of incubation in blood, when most of the yeast cells are phagocytized. In *C. albicans*, a high level of different transcripts involved in protein synthesis was also detected at the beginning of the incubation in blood, but the mRNA level of these genes decreased during the incubation [19], indicating that growth initiation occurred only in the early stages. This suggests that *C. albicans* has a faster response to adapt to blood environment than *S. cerevisiae*.

We observed that the induction of genes involved in aminoacid biosynthetic pathway is present during the middle and the later time course in the virulent strains, while in non-virulent strains they appear later or don’t appear. Also, following exposure to human neutrophils or cultured macrophages, *C. albicans* populations upregulate amino acid biosynthetic genes [20,26]. Rubin-Bejerano et al. [26] observed an induction of these pathways after yeast cells were ingested by neutrophils, but it was not present when yeast cells were ingested by human monocytes. It may suggest that the microenvironment in the phagosome inside the neutrophil is deficient in amino acids and it generates a rapid response from the virulent *S. cerevisiae* strains. However, non-virulent strains are slower or less efficient in the adaptation to this microenvironment, decreasing their possibilities to survive. In addition, the methionine and arginine biosynthetic genes are not induced when *S. cerevisiae* is phagocytized by the murine macrophage-like cell line J774A [27]. Additionally, Kingsbury et al. [28] revealed the relevance of amino acid biosynthesis for yeast survival in murine host; however processes important for sensing and responding to quality and concentration of nitrogen compounds were not required for yeast survival *in vivo*, indicating that yeast can use a variety of nitrogen sources in these conditions. The pyridoxine metabolic process genes were downregulated in the virulent strains with regards to non-virulent strains. Padilla et al. [29] observed that these genes were expressed under nutrient limitation, so it may reflect that strains 60 and D14 were not exposed to a limitation of specific nutrients, such as nitrogen.

The glyoxylate cycle is induced upon phagocytes ingestion of the bacteria *Mycobacterium tuberculosis*[30] and other fungi such as *C. neoformans*[31], *C. albicans*[27], *Leptosphaeria maculans*[32] and *S. cerevisiae*[27]. This shift in metabolism has been interpreted as a response to the glucose-poor environment of the macrophage, and the ability to make that shift appears to contribute to the virulence of some pathogens. However, in our condition using complex blood medium, the genes encoding the principal enzymes of the glyoxylate cycle, isocitrate lyase (*ICL1*) and malate synthase (*MLS1*), were downregulated during the time course, although this repression was less strong at the later stage in strains D14 and 60. This suggests that yeast cells have access to glucose during almost all the experiment. The fact that *icl1*Δ mutants were only slightly deficient *in vivo*[33] suggests that the glyoxylate cycle has a minor contribution to *S. cerevisiae* fitness *in vivo*. The *ICL1* gene of both *S. cerevisiae* and *C. albicans* has recently been shown to be substantially induced upon exposure to macrophages *in vitro*[27,34]. However, these experiments were performed with murine macrophages in cell culture medium, where glucose concentration may be different from blood. Furthermore, a *C. albicans icl1*Δ/*icl1*Δ mutant showed a substantial reduction in virulence [27], while the same mutant was not attenuated in survival in blood [20], suggesting that the *ICL1* gene may play a general role when *C. albicans* has left the bloodstream. All these data suggest that the *ICL1* gene may play also a general role in *S. cerevisiae* in human infections but after yeast cells has left the bloodstream.

When we compared the transcriptomes of virulent and control strains, we observed several specific groups of genes that may explain the pathogenic nature of the virulent strains. An interesting functional group of up-regulated genes during blood incubation was Cell redox homeostasis. We confirmed that this increased oxidative stress response correlates with phenotypical advantage of virulent strains in pro-oxidant environments since they have significantly much higher survival in the presence of high concentrations of H_2_O_2_. Furthermore, they survive to oxidative burst attack from blood cells significantly better than control strain W303 and at similar levels that pathogenic *C. albicans*. The decreased survival of *YAP1* mutant strain in human blood incubations highlights the importance of the genes included in the Yap1p regulon in determining the virulence of *S. cerevisiae* strains.

Since we have used a molecular transcriptomic approach and human blood media, it was difficult to use several virulent and non-virulent strains, in order to obtain a broad comparative view. However, Diezmann and Dietrich [35] compared hundreds of clinical isolates due to the use of easy tractable phenotypical assays. In concordance with our results, Diezmann and Dietrich [35] showed that *S. cerevisiae* clinical isolates were more resistant to oxidative stress. This data suggest a correlation between survival in oxidative stress and yeast pathogenicity and strongly supports our data. Macrophages, neutrophils and other phagocytic cells generate potent reactive oxygen and nitrogen species (ROS and RNS), which are toxic to most fungal pathogens, causing damage to DNA, proteins and lipids [36]. Fungal pathogens display different degrees of resistance to the reactive oxygen and nitrogen species used by human cells to counteract infection [37]. Fungal resistance to ROS offers protection from oxidative host defenses and is undoubtedly an advantageous pathobiological property [38,39]. It is worth mentioning that several genes belonging to the thioredoxin system were upregulated, which suggests its implication in yeast defense against blood defenses. Indeed, the thioredoxin system of *C. albicans* has been shown to be expressed during growth in human blood or mucosal tissue [19,20,40], indicating that ability to respond to oxidative stress might be crucial in the early stages of systemic *C. albicans* infections. Also, *TRX1* is necessary for survival of *C. neoformans* in the oxidative environment of macrophages and important for virulence of this fungal pathogen [41]. *TSA2* and *GPX2* genes have been shown to be induced in *S. cerevisiae* strains when exposed to neutrophils [26], and a clear antioxidant response has been observed. Fradin et al. [20] demonstrated that neutrophils play a key role in bloodstream infections with *C. albicans*. This observation is in line with the high susceptibility of neutropenic patients (deficient in these immune cells) to disseminated candidiasis [42,43].

In conclusion, this work supports the view that oxidative stress response of *S. cerevisiae* strains has a special importance for survival in blood, having a high impact in determining their virulence. This characteristic can help these yeasts to prevail in immunocompromised patients and cause systemic infections.

## Conclusions

In the present work we have found significant differences at molecular level between *S. cerevisiae* non-virulent strains and virulent clinical strains with remarkable capabilities of infect and even produce mice death. Transcription analysis in blood incubations pointed out an enhanced oxidative stress response of virulent strains that is reflected in an increased ability to survive in the presence of high concentrations of the oxidant H_2_O_2_. Indeed, they are able to survive to a similar extent than pathogenic *C. albicans* strain in blood incubations. These data suggest that *S. cerevisiae* virulent strains are able to extend the survival after the macrophage oxidative burst attack, having more chances to persist and perform systemic infections.

## Methods

### Strains and media

Several *S. cerevisiae* isolates were used in this work: a clinical isolate from vagina (isolate 60) [7], a brewer’s strain isolated from a commercial nutritional complement product (D14) [18], and two control strains: (one laboratory strain W303 (MATa; ura3-52; trp1Δ2; leu2-3,112; his3-11; ade2-1; can1-100) and a wine strain CECT 10431) [18]. Isolate 60 and D14 were chosen from a virulent yeast collection obtained from several sources because they have the highest levels in virulence factors and because they were able to survive and colonize the brain of immunocompetent mice after blood infections and cause mice death, showing increased adherence ability [3,18]. Also, they were phagocytosed by macrophages with less efficiency than CECT 10431 [18]. The natural wine strain CECT 10431 was used as negative control because it is unable to proliferate in any organ after its intravenous inoculation in murine systems [3,18]. *Saccharomyces cerevisiae* var. *boulardii* commercial preparations also known as Ultralevure were used in blood survival experiments. *C. albicans* SC5314 [44] was used as a positive control yeast in blood incubations. *S. cerevisiae* and *C. albicans* strains were cultured in YPD medium (1.0% yeast extract, 1.0% Bacto-peptone and 2.0% glucose) prior to blood incubations.

The mutant D14Δyap1 strain was obtained using a PCR-based gene-replacement strategy using a deletion cassette containing geneticin resistance amplified from pUG6 plasmid [44]. The primers used are included in the Additional file 1: Table S6. These primers had between 40 homologous bases to the ends of the gene coding sequence. The amplified PCR product was used for yeast modification by homologous recombination following yeast transformation using the lithium acetate procedure. PCR reactions using plasmid pUG6 as template were as follows: 2 min at 94°C; 30 cycles of 15 s at 94°C, 30 s at 50°C, 2 min at 72°C and 5 min at 72°C. Cells were transformed with the PCR product using the LiAc protocol (http://home.cc.umanitoba.ca/~gietz/). After heat shock, cells were incubated for 3 hours in YPD liquid medium at 30°C. Finally, transformed cells were selected on YPD plates with geneticin (200 mg/L). Geneticin resistance was removed by transforming with pSH47, a plasmid containing Cre recombinase, inducing in galactose for 24 h and selecting colonies without geneticin resistance. Since D14 strain is diploid, the second *YAP1* gene deletion was performed with the same procedure but using a replacement cassette containing hygromycin B resistance amplified from pAG32 plasmid [45]. The *YAP1* gene deletion was confirmed by PCR analysis and resistance to H_2_O_2_. The strain D14YAP1 was obtained by transforming D14Δyap1 strain with a plasmid containing a wild type copy of *YAP1* (pYAP1) and selecting in YPD with 200 μg/mL of G418 (Sigma). The plasmid obtained from Kuge et al. [46] was marker swapped for KANMX by homologous recombination using a cassette obtained by PCR and using pUG6 plasmid as a template.

### Human blood incubations

Yeast cells were precultured overnight in YPD medium at 30°C. Cells were counted and preadapted in PBS buffer at a density of 2.5 x 10^9^ cells/ml (corresponding to two cells per leukocyte) incubating 30 min at 37°C before inoculation in human blood [22]. Human peripheral venous blood was obtained with signed informed consent under protocols approved by the bioethics commission of Centro Superior de Investigaciones Científicas. Human peripheral venous blood was collected from healthy volunteers by venipuncture using ammonium heparin syringes (Monovette®, Sarstedt). Preadapted cell suspension (100 μl) was inoculated into 25 ml of fresh whole human blood and incubated for 0, 15, 30 and 60 min at 37°C in semi-aerated cultivation flasks. At each time point, 2 volumes of 0.5% Triton X-100 were added to break blood cells. Then the cells and blood cells debris were collected by centrifugation at 1300 x g for 3 min at 4°C. The pellet with yeast and blood cells debris was transferred to a tube with 1 ml of cold water. In order to separate yeast cells, a fast centrifugation pulse (70 x g for 15 seconds) was performed. This treatment precipitated blood cells debris maintaining only yeast cells in the supernatant. This supernatant was centrifuged at 1300 x g for 2 minutes at 4°C and the resulting pellet was immediately frozen in liquid nitrogen. Control samples (time point 0) were treated exactly the same way as the other samples to exclude variations due sample manipulation in the transcriptomic data.

### RNA extraction and labeling

Frozen cells were lysed and homogenized by vortexing in LETS buffer (10 mM Tris pH 7.4, 10 mM lithium-EDTA, 100 mM lithium chloride, 1.0% lithium lauryl sulfate) with acid-washed glass beads (0.4 – 0.6 mm; Sigma-Aldrich) for 3 min, and total RNA was extracted using phenol:chloroform RNA method [47]. mRNA was amplified using Low RNA Input Fluorescent Linear Amplification Kit (Agilent Technologies) according to the manufacturer’s instructions. The amplification is divided in two steps: a first step to synthesize cDNA using oligo (dT) primer and a second step to synthesize cRNA. The amplified cRNA obtained was purified using RNeasy kit (Qiagen) following the instructions. cDNA for transcriptional profiling was produced by incubating 1.5 μg (16.5 μl) amplified cRNA with 1 μl (0.4 μg/μl) random hexamers (Agilent) at 70°C for 5 min. After adding 1x first strand buffer, 12 mM DTT, 500 μM dATP, 500 μM dCTP, 500 μM dGTP, 160 μM dTTP (Fermentas), 330 μM aminoallyl- dUTP (Fermentas), 10 U ribonuclease inhibitor (Invitrogen) and 400 U SuperScript III reverse transcriptase (Invitrogen) the mix was incubated for 16 hours at 50°C. cDNA obtained was purified using MinElute PCR Purification kit (Qiagen). Labeling reaction was produced incubating 1.5-2 μg of aminoallyl-cDNA with 3 μl of Cy3 and Cy5 fluorophores (Amersham) at basic pH (0.2 M Na_2_CO_3_, pH 9) for 2 hours at room temperature. Labeled cDNA was purified using the same kit mentioned above.

### Microarray hybridization and analysis

For transcript profiling, *S. cerevisiae* microarrays were used, which were synthesized in duplicate on glass by Microarray Center of University Health Network (Canada), comprising 6,240 yeast ORFs. Microarrays were prehybridized for 45 min at 42°C in 3 x SSC (0.15 M NaCl and 0.015 M sodium citrate), 0.1% (w/v) SDS and 0.1 mg/ml BSA. Prehybridized microarrays were washed twice with water, once with isopropanol and then dried by centrifugation. Before hybridization, equal quantities of Cy5- and Cy3-labelled cDNA, 50% (v/v) formamide, 5 x SSC and 0.1% (w/v) SDS were mixed, made up to a final volume of 60 μl with water and denatured for 1 min at 95°C. The mixture was applied to the prehybridized microarrays, covered with a Hybri-slip (60 x 24 mm; Sigma-Aldrich) and hybridization took place for 16 hours in a humidified chamber in a water bath at 42°C. Hybridized microarrays were washed for 5 min at 42°C in 2 x SSC, 0.1% (w/v) SDS, for 20 min at room temperatures in 0.1 x SSC, 0.1% (w/v) SDS, for 6 min at room temperatures in 0.1 x SSC, and for 1 min at room temperatures in 0.01 x SSC. Hybridized microarrays were dried by centrifugation. After washing, the microarrays were immediately scanned with an Axon 4100A scanner at a resolution of 10 μ m and data were analyzed using the GenePix Pro 6.1 software package (Axon Instruments). Hybridization specificity was assured because no signal was observed in control spots with bacterial or plants unspecific DNA. Data were ratio-based normalized and processed using Acuity 4.0 (Axon Instruments). False Discovery Rate (FDR) of 5% was used to identify statistically significant data. A cut-off rate of >2-fold was used to select for induced genes. Functional group analysis was done using GOstat, DAVID Database and GO term finder (SGD database) online applications selecting for Bonferroni corrected *p*-values < 0.05. mRNA level of selected genes was confirmed by qRT-PCR. The microarray transcriptomic data presented correspond to the average of three biological replicates (separate cultures on different days) including a dye swap. The microarray data was deposited in ArrayExpress database with the accession number E-TABM-1131.

### qRT-PCR

To validate microarrays results, amplified RNA was converted to cDNA and mRNA level of seven genes, in different strains and conditions, was quantified by qRT-PCR. 1 μg of RNA was mixed with 0.5 mM dNTPs, 50 pmol Oligo(dT) in 10 μl. The mix was heated to 65°C for 5 min and chilled on ice. Then 10 mM DTT, 50U RNAse inhibitor (Invitrogen), 1x First Strand Buffer (Invitrogen) and water to 20 μl were added to the mix and it was incubated 2 min at room temp. After adding 500 U Superscript III (Invitrogen), samples were incubated at 42°C for 50 min and reaction was inactivated 15 min at 70°C. Agarose gel electrophoresis was done to check nucleic acid contaminations. qRT-PCR was performed with gene-specific primers (200 nM) (Additional file 1: Table S6) in a 20 μl reaction, using the Light Cycler Fast Start DNA Master PLUS SYBR green (Roche Applied Science, Germany) in a LightCycler® 2.0 System (Roche Applied Science, Germany) device. All samples were processed for melting curve analysis, amplification efficiency and DNA concentration determination using LightCycler® 2.0 System. A mix of all samples and serial dilutions (10^-1^ to 10^-5^) were used as standard curve.

### Survival during oxidative stress

Strains were grown overnight in YPD at 30°C. After adjusting to 1 x 10^6^ cells/ml in PBS, 6 mM H_2_O_2_ (Panreac) were added and samples were incubated for one hour at 30°C with shaking. Dilutions 10^-2^ to 10^-4^ were done and 100 μl from these dilutions were spread on YPD plates, incubated for 48 h at 30°C and cfu were counted. Survival was calculated as the ratio of treated over untreated cells (time point 0 min). To compare resistance to oxidative stress of D14, D14YAP1 and D14ΔYap1 strains cells were patched on YPD or YPD with 2.5 mM H_2_O_2_ plates and let them grow for 48 h. Each strain was tested at least in three independent experiments. Data are represented as averages ± standard error and statistical significance was determined using Student’s *t* test (see Additional file 1: Table S5).

### Survival assay in human blood

To investigate the survival of yeast strains in blood, *C. albicans* SC5314 and four *S. cerevisiae* strains were assayed. Yeast strains were grown overnight in YPD medium at 30°C. The cells were washed once and suspended in PBS buffer (Phosphate-buffered saline: 150 mM NaCl, 16 mM Na_2_HPO_4_, 4 mM NaH_2_PO_4_, pH 7.4) at a density of 5 x 10^7^ cells/ml. Human peripheral venous blood was collected from healthy volunteers by venipuncture using ammonium heparin syringes (Monovette®, Sarstedt). Yeast cells were inoculated (1:1 ratio of yeast: leukocytes) in blood and incubated for 10 and 60 min at 37°C. At each time point, 100 μl of 10^-2^ to 10^-4^dilutions were spread on YPD plates and incubated for 48 h at 30°C. Colony-forming units (cfu) were counted and percentages of survival determined as follows: (cfu/cfu_plasma_) x 100. Each strain was tested three times. Data were represented as averages ± standard error.

## Competing interests

The authors declare that they have no competing interests.

## Authors’ contributions

SLl carried out transcriptomics experiments and data analysis, blood survival assays and drafted the manuscript. RPT carried out transcriptomics experiments and data analysis, blood survival assays, oxidative stress assays and mutant construction. AH helped with blood survival assays. BH supervised blood survival assays and corrected the manuscript. LJ participated in the design of the study and supported the study. AQ and MTFE participated in the design of the study and corrected the manuscript. RPT conceived the study and participated in the design of the study and drafted the manuscript. All authors read and approved the final manuscript.

## Supplementary Material

Additional file 1 **Table S1.** Genes and statistical significance (p-values) of the functional groups expressed at 15, 30 and 60 min of incubation in blood in virulent strains (60 and D14) and non-virulent (CECT 10.431 and W303). **Table S2.** Genes and statistical significance (p-values) of the functional groups repressed at 30 min of incubation in blood in virulent strains (60 and D14) and non-virulent (CECT 10431 and W303). **Table S4.** qRT-PCR validation of transcriptomic data. **Table S5.** Statistical pairwise comparisons of Figure 2. **Table 6.** Primers used in this study. Click here for file

Additional file 2 **Table S3. **Complete microarray dataset. Click here for file
