# Supplementary material for: Transcriptomics in human blood incubation reveals the importance of oxidative stress response in Saccharomyces cerevisiae clinical strains
Source: BMC Genomics. 2012 Aug 23;13:419. doi: 10.1186/1471-2164-13-419 (PMC3483181; doi:10.1186/1471-2164-13-419)
Supplement: Additional file 1 — Table S1. Genes and statistical significance (p-values) of the functional groups expressed at 15, 30 and 60 min of incubation in blood in virulent strains (60 and D14) and non-virulent (CECT 10.431 and W303). Table S2. Genes and statistical significance (p-values) of the functional groups repressed at 30 min of incubation in blood in virulent strains (60 and D14) and non-virulent (CECT 10431 and W303). Table S4. qRT-PCR validation of transcriptomic data. Table S5. Statistical pairwise comparisons of Figure 2. Table 6. Primers used in this study. [file 1471-2164-13-419-S1.docx]

**SUPPLEMENTARY TABLES**

**Supplementary Table 1**. Genes and statistical significance (p-values) of the functional groups expressed at 15, 30 and 60 min of incubation in blood in virulent strains (60 and D14) and non-virulent (CECT 10.431 and W303).

|  | p-value | Genes | |
| --- | --- | --- | --- |
| **Up-regulated 15’** |  | | |
| BASE-EXCISION REPAIR | 0.01224 | | *POL30, POL31, RAD27, APN1, OGG1* |
| VACUOLE ORGANIZATION | 0.00489 | | *CMD1, VPS3, CUP5, VTC1, SFK1* |
| XENOBIOTIC TRANSPORTER | 0.01990 | | *PDR5, SNQ2, PDR12* |
| **Up-regulated 30’** |  |  |  |
| AMINO ACID BIOSYNTHETIC PROCESS | 0.00051 | | *ARO4/ ARO3/ LYS4/ HIS1/ ARG5,6/ SER3/ILV1/ SER33/ HIS5/ LYS1/ TRP3/ ARG1/ ARG8/ LEU9/ ORT1/ SAM4/* |
| AMINE BIOSYNTHETIC PROCESS | 0.00143 | |  |
| NITROGEN COMPOUND BIOSYNTHETIC PROCESS | 0.00162 | |  |
| AMINO ACID METABOLIC PROCESS | 0.00855 | | *ADH5/ ARO4/ ARO3/ LYS4/ HIS1/ ARG5,6/ SER3/ ILV1/ADH4/ SER33/ HIS5/ LYS1/ URA2/ TRP3/ MSE1/ ARG1/ ARG8/ LEU9/ ORT1/ SAM4/* |
| PHOSPHATASE ACTIVITY | 1.78x10^-5^ | | *PTC3/ PHO3/ PHO5/ DPP1/ LPP1/ SDT1/ PHO12/ INP51/ SAP185/ SAC1/ PPZ1/ TSL1/ YMR087WTPS3/ FCP1/ YNL217W* |
| TRANSMEMBRANE TRANSPORTER ACTIVITY | 0.01608 | | *CTP1/ GGC1/ ATP17/ GNP1/ PIC2/ HXT10/ VPS73/ ZRT1/ TPO2/ TNA1/ AVT1/ VMA5/ ZRT3/ YBT1/ AQY2/ SMF3/ ZRT2/ ATR1/ ITR2/ ORT1/ ODC2/ PDR12/ YMC1/ ANT1/ TPO3/* |
| VACUOLE ORGANIZATION | 0.00175 | | *VID24/ MD1/ VTC1/ VPS45/ TRX2/ SFK1/ TRX1/ VAC7/ TPM1/ VTC3/* |
| **Up-regulated 60’** |  |  |  |
| AMINO ACID BIOSYNTHETIC PROCESS | 0.00086 | | *ARO4/ LYS4/ HIS1/ ARG5,6/ SER3/ILV1/ HIS5/ LYS1/ ARG1/ ARG8/ SAM4/* |
| AMINE BIOSYNTHETIC PROCESS | 0.00179 | |  |
| NITROGEN COMPOUND BIOSYNTHETIC PROCESS | 0.00196 | |  |
| AMINO ACID METABOLIC PROCESS | 0.00636 | | *ARO4/ LYS4/:HIS1/ ARG5,6/ SER3/ ILV1/ HIS5/ LYS1/ URA2/ MSE1/ ARG1/ ARG8/ SAM4/* |
| CELL REDOX HOMEOSTASIS | 0.00998 | | *TTR1/ TRX2/ TRX1/ TSA1/ GLR1* |
| PHOSPHATASE ACTIVITY | 0.00604 | | *PHO3/ PHO5/ PHO12/* |

**Supplementary Table 2**. Genes and statistical significance (p-values) of the functional groups repressed at 30 min of incubation in blood in virulent strains (60 and D14) and non-virulent (CECT 10431 and W303).

|  | p-value | Genes | |
| --- | --- | --- | --- |
| **Down-regulated 30’** |  | | |
| PYRIDOXINE METABOLIC PROCESS | 0.00694 | | *SNZ3/ SNZ2/ SNO2* |

**Supplementary Table 4**. qRT-PCR validation of transcriptomic data

| Gene | Strain | Time | Microarrays | qRT-PCR | Microarray-qRT-PCR  Difference (%)^a^ |
| --- | --- | --- | --- | --- | --- |
| *PHO5* | 60 | 60' | 2,85 | 2,97 | 1,86 |
| *GFA1* | 60 | 60' | 1,39 | 1,34 | 1,98 |
| *PHO3* | 60 | 60' | 2,30 | 2,16 | 2,87 |
| *PHO12* | 60 | 60' | 3,18 | 2,98 | 3,18 |
| *PSA1* | D14 | 60' | 3,35 | 3,07 | 4,10 |
| *YNL217* | 60 | 60' | 1,64 | 1,34 | 9,09 |
| *TRX1* | 60 | 60' | 1,73 | 1,35 | 11,07 |
| *GFA1* | D14 | 60' | 1,11 | 0,80 | 14,11 |
| *PSA1* | 60 | 60' | 3,81 | 2,70 | 14,52 |
| *YNL217* | D14 | 60' | 1,16 | 1,61 | 19,19 |

^a^Microarray data was divided by qRT-PCR and multiplied by 100. Absolute numbers are shown

**Supplementary Table 5**. Statistical pairwise comparisons of Figure 2

|  | CECT10431 | W303 | 60 | D14 |
| --- | --- | --- | --- | --- |
| CECT10431 | - | ns | * | * |
| W303 | - | - | ** | ** |
| 60 | - | - | - | ns |
| D14 | - | - | - | - |

ns: not significant; * P<0.0005;** P<0.00005

**Supplementary Table 6**. Primers used in this study

| Name | Use | Forward | Reverse |
| --- | --- | --- | --- |
| Δyap1 | *YAP1* deletion | ATGAGTGTGTCTACCGCCAAGAG  GTCGCTGGATGTCGTTTCTCCGG  GTTCCGTACGCTGCAGGTCGAC | TTAGTTCATATGCTTATTCAAAGC  TAATTGAACGTCTTCTGCATTGAT  GACACTAGTGGATCTGATATC |
| *YAP1*-A1 | *YAP1* deletion | ACTTGTGCATGAACACGAGCC | - |
| *YAP1*-A2 | *YAP1* deletion | GGAGCCGAATGACTTGGATC | - |
| *YAP1*-A3 | *YAP1* deletion | CTTTCGGTTACCCAGTTTTCC | - |
| *TRX1* | qPCR | CGCTATGCCAACTTTGCTT | CTTAATAGCCGCTGGGTTG |
| *PHO5* | qPCR | TGGTCCAGGGTTCTCTTGT | TACTGACGCTGCTGACGTT |
| *PHO3* | qPCR | CGGAGAGTTAGCCGATGTT | CGCCAGGGAAAGAGAAGTA |
| *PHO12* | qPCR | TGGTCCAGGGTTCTCTTGT | TACTGACGCTGCTGACGTT |
| *PSA1* | qPCR | GTGCAACTCCACCATCAAG | CAGTGACACCTTCCAAACG |
| *YNL217w* | qPCR | GTGGGACAAATACGGAGAC | GGTTTAGTCCCATGGATGC |
| *GFA1* | qPCR | CTGCTCTGGAAGGTGCTTT | CAAGACACCGTGCTTCAAC |
| Ura:KAN | Marker swap | ATGTCGAAAGCTACATATA  AGGAACGTGCTGCTACTCA  TCCGTACGCTGCAGGTCGAC | TTAGTTTTGCTGGCCGCATC  TTCTCAAATATGCTTCCCAG  CCACTAGTGGATCTGATC |
